# Comprehensive Noise Modeling of Piezoelectric Charge Accelerometer with Signal Conditioning Circuit

**DOI:** 10.3390/mi15020283

**Published:** 2024-02-17

**Authors:** Ghulam Ali, Faisal Mohd-Yasin

**Affiliations:** School of Engineering and Built Environment, Griffith University, Nathan, QLD 4111, Australia; ghulam.ali@griffithuni.edu.au

**Keywords:** noise, modeling, piezoelectric, accelerometer, charge amplifier

## Abstract

This paper reports on noise modeling of a piezoelectric charge accelerometer with a signal conditioning circuit. The charge output is converted into voltage and amplified using a JFET operational amplifier that has high input resistance and low noise. The noise sources in the whole system include electrical and mechanical thermal noises of the accelerometer, thermal noises of resistors, and voltage and current noises of the operational amplifier. Noise gain of each source is derived from small signal circuit analysis. It is found that the feedback resistor of the operational amplifier is a major source of noise in low frequencies, whereas electrical thermal noise of the accelerometer dominates the rest of spectrum. This method can be used to pair a highly sensitive sensor with a single JFET operational amplifier instead of a multi-stage signal conditioning circuit.

## 1. Introduction

Piezoelectric charge accelerometers have extensively been utilized in vibration and shock measurements in industrial and scientific applications [1] since they offer wide bandwidth, broad temperature, dynamic range, high sensitivity, and linearity [2,3,4,5,6]. The applications include but are not limited to seismic monitoring, medical instrument installation, automobile vibration, machine monitoring, as well as high-temperature and high-radiation environments. These accelerometers typically utilize a piezoelectric sensing element that produces the electric charges when acceleration is detected [7,8]. As the amount of generated charges is quite small, a signal conditioning circuit is required to amplify the output [9]. In some applications, this circuit is also used to perform analog voltage scaling, frequency filtering, and conversion from analog to digital signals.

Electrically, a piezoelectric accelerometer is a sensor whose capacitance is dependent on the relative permittivity, distances between plates, and the overlapping area [9]. Because of their small capacitances and low piezoelectric material losses, these sensors have high output impedance in the range of MΩ to GΩ [10,11]. In order to avoid a loading effect, an extremely high impedance circuit must be utilized at the input stage of the signal conditioning system [12,13,14,15]. After that, the signal from the sensor is fed to the charge amplifier to convert and amplify the charges [16]. In terms of practical measurement, the common practice is to target a high signal-to-noise ratio implementation to avoid issues with noise signals. However, this technique does not work for all situations. When an ultra-sensitive detection of the input signals is required, the noise sources from the sensor and the signal conditioning circuitry need to be properly accounted for.

There have been a lot of works in reducing the noise of the signal conditioning circuit of any sensor. In the case of the piezoelectric accelerometer, the most prominent works are contributed by Felix Levinzon. His 2008 paper [11] discussed the ultra-low noise amplifier that consists of five stages. The first one is the JFET input stage to provide high input impedance. The next four stages are used to provide high gain. These are made of operational amplifier, FETs, and BJT. In terms of the noise analysis, Levinzon only considered the noise sources from the JFET input stage; its noise density is engineered to be much larger than the noise contributions of the subsequent amplification stages.

In this paper, we perform comprehensive noise modeling of the piezoelectric charge accelerometer and its signal conditioning circuit. We propose the use of a single stage JFET operational amplifier, as this chip is capable of providing high input impedance as well as large gain. Our proposed system consists of eight noise sources, including the electrical and mechanical thermal noises of the accelerometer, the thermal noises of the resistors, and the voltage and current noises of the operational amplifier. We employ small signal circuit analysis to come up with the noise equations. A similar method was performed by Durdaut et al. to analyze the noise sources of a magnetoelectric sensor but with a more complex signal conditioning circuit [10]. The organization of the rest of this paper is as follows. The circuit and noise modeling of the piezoelectric charge accelerometer with JFET operational amplifier are presented in Section 2. Section 3 will discuss the results and discussion, while Section 4 offers several practical considerations to effectively make use of the proposed model. Section 5 concludes this paper.

## 2. Methodology

This modeling work is implemented in two stages. In Section 2.1, the equivalent circuits for the piezoelectric accelerometer and charge amplifier are simulated in LTSpice using parameters from published works and a datasheet of JFET operational amplifier. This simulation work is needed to validate the targeted gain and frequency responses. 

In Section 2.2, we first identify all the noise sources inside the accelerometer and amplifier. After that, the noise gain equation is derived based on the small signal circuit analysis for each source. In total, seven equivalent circuits have been sketched to complete the derivations. Finally, the noise density for all sources is calculated.

### 2.1. Circuit Modeling

**Figure 1:**
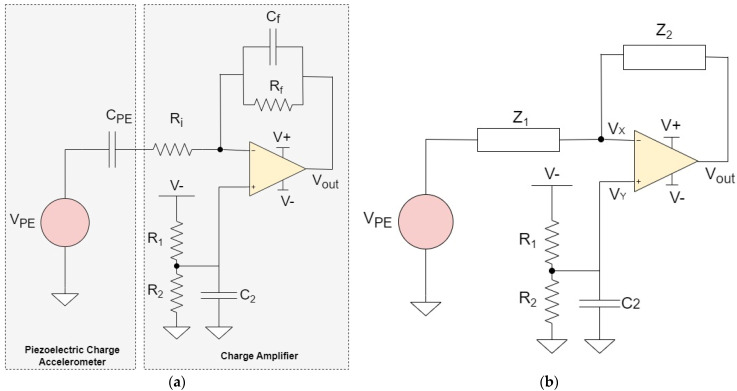
(**a**) Circuit of piezoelectric accelerometer and charge amplifier. (**b**) Circuit of Figure 1a redrawn to indicate the voltages $V_{X}\;and\;V_{Y}$ and the impedances Z_1_ and Z_2_.

Figure 1a shows the complete system, i.e., piezoelectric charge accelerometer and the charge amplifier. The accelerometer is modeled with a capacitor C_PE_ in series with a voltage source *V*_PE_ [16,17], which is then fed to the negative input of the operational amplifier [18]. The key parameters for the accelerometer are taken from the Levinzon’s prototype [19] and are listed in Table 1. Since the charge sensitivity *Q*_PE_ is 1 pC/g, the piezoelectric accelerometer is modeled as a 10 mV voltage source in series with a 100 pF capacitor. Both values will produce 1 pC of charges in the circuit simulation. This value is chosen to simulate the sensitive detection of small acceleration.

The schematic of the signal conditioning circuit is shown in Figure 1a. It is essentially a basic operational-based charge amplifier circuit. We have chosen LT1169 from Linear Technology [18] to serve as the operational amplifier due to its low voltage noise (6 nV/√Hz), low current noise (1 fA/√Hz), high input resistance (10^13^ Ω), and high open-loop gain (1.2 × 10^6^). The −3 dB lower cutoff frequency of the charge amplifier is dependent on the feedback resistance R_f_ and capacitance C_f_ as follows:

(1) $$f_{L}=\frac{1}{2\pi R_{f}C_{f}}$$

whereas the −3 dB upper cutoff frequency is dependent on the input resistance R_i_ and piezoelectric capacitance C_PE_ as follows:

(2) $$f_{H}=\frac{1}{2\pi R_{i}C_{PE}}$$

The resonant frequency of the accelerometer is 30 KHz and the operating range is 0.5 Hz to 10 KHz. The corresponding values of the amplifier parameters to meet this bandwidth requirement are given in Table 2. The resistors R_1_ and R_2_ are added to compensate for the offset voltage at the input terminals of the operational amplifier. The value of the open-loop gain ‘Aop’ is taken from the product datasheet [18]. In order to derive the output and impedance equations, we redraw Figure 1a to show the voltages $V_{X}\;and\;V_{Y}$ and the impedances Z_1_ and Z_2_. The redrawn circuit is shown in Figure 1b. Z_1_ and Z_2_ are defined as:

(3) $$Z_{1}=R_{i}+{\left(sC_{PE}\right)}^{-1}=\frac{s{R_{i}C}_{PE}+1}{sC_{PE}}$$

(4) $$Z_{2}=R_{f}||{\left(sC_{f}\right)}^{-1}=\frac{R_{f}}{s{R_{f}C}_{f}+1}$$

where ‘s’ shows the Laplace transform. $V_{Y}$ can be obtained from the voltage divider as follows:

(5) $$V_{Y}=\frac{R_{2}||{\left(sC_{2}\right)}^{-1}}{R_{1}+R_{2}||{\left(sC_{2}\right)}^{-1}}\;V_{-}=\frac{R_{2}}{R_{1}+R_{2}+sR_{1}R_{2}C_{2}}\;V_{-}$$

If $A_{op}$ is the open-loop gain of the Op-Amp, then the output voltage can be written as follows:

(6) $${(V}_{Y}-V_{X})A_{op}=V_{out}$$

(7) $$⟹\;\;V_{X}=V_{Y}-\frac{V_{out}}{A_{op}}$$

KCL at node $V_{X}$ gives:

(8) $$\frac{V_{X}-V_{PE}}{Z_{1}}=-\frac{V_{X}-V_{out}}{Z_{2}}$$

(9) $$⟹\;V_{X}(\frac{Z_{1}+Z_{2}}{Z_{1}Z_{2}})-\frac{V_{out}}{Z_{2}}=\frac{V_{PE}}{Z_{1}}$$

By using Equation (7) in (9) and rearranging, we obtain:

(10) $$V_{out}=-\frac{{A_{op}Z}_{2}}{{{(A}_{op}+1)Z}_{1}+Z_{2}}V_{PE}+V_{Y}(\frac{A_{op}(Z_{1}+Z_{2})}{{{(A}_{op}+1)Z}_{1}+Z_{2}})$$

By using Equation (5) in (10), we obtain the output voltage equation as:

(11) $$V_{out}=-\frac{{A_{op}Z}_{2}}{{{(A}_{op}+1)Z}_{1}+Z_{2}}V_{PE}+(\frac{R_{2}}{R_{1}+R_{2}+sR_{1}R_{2}C_{2}})(\frac{{A_{op}(Z}_{1}+Z_{2})}{{{(A}_{op}+1)Z}_{1}+Z_{2}})V_{-}$$

### 2.2. Noise Modeling

**Figure 2:**
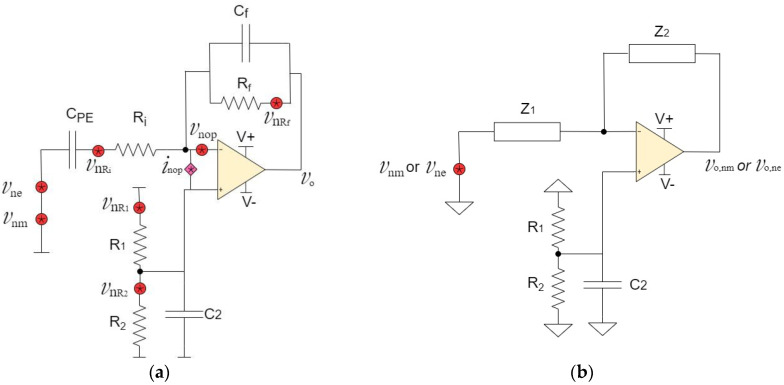

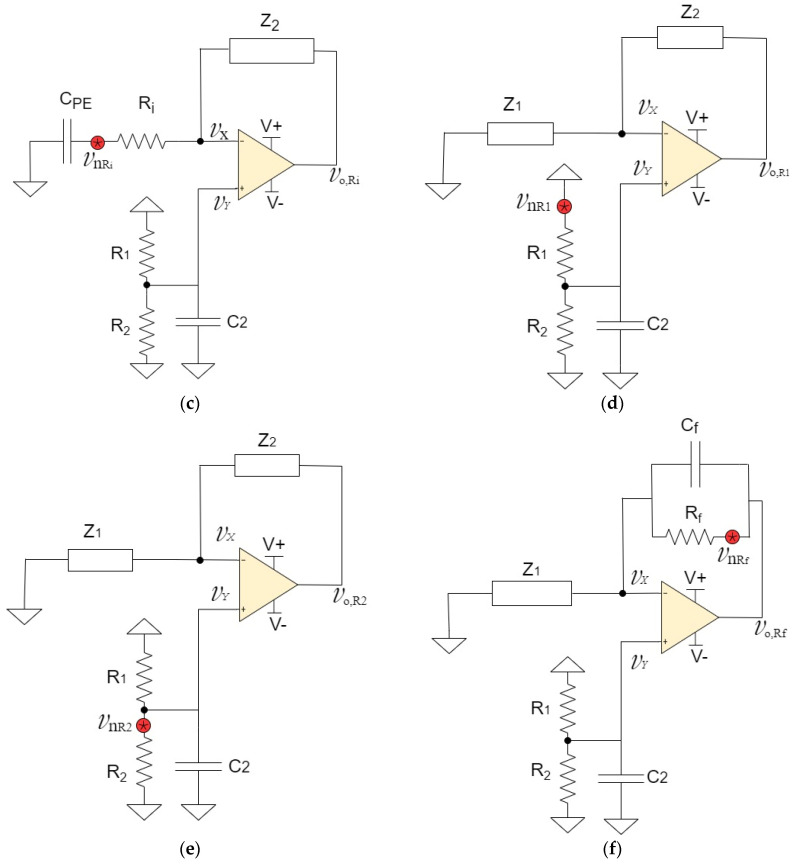

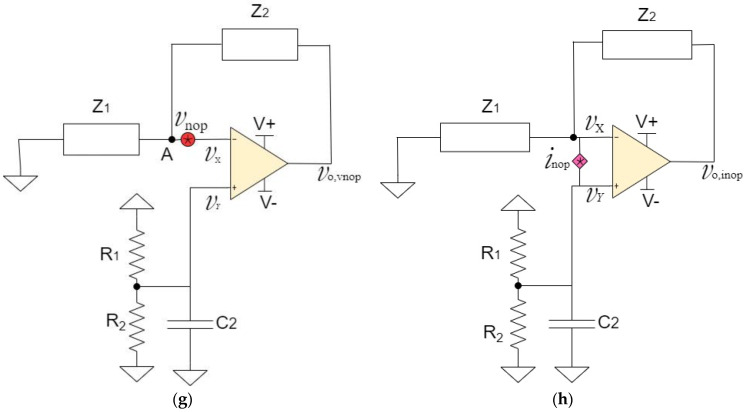
(**a**) Noise sources within the piezoelectric accelerometer and charge amplifier circuit are denoted by red dots. (**b**) Circuit of Figure 1b redrawn for the derivation of $G_{nm}$ and $G_{ne}.\;$(**c**) Circuit of Figure 1b redrawn for the derivation of $G_{nRi}.\;$(**d**) Circuit of Figure 1b redrawn for the derivation of $G_{nR1}.\;$(**e**) Circuit of Figure 1b redrawn for the derivation of $G_{nR2}.\;$(**f**) Circuit of Figure 1b redrawn for the derivation of $G_{nRf}.\;$(**g**) Circuit of Figure 1b redrawn for the derivation of $G_{nop}.\;$(**h**) Circuit of Figure 1b redrawn for the derivation of $G_{inop}$.

Figure 2a shows all the noise sources in the accelerometer and the charge amplifier. The noise sources within this system include electrical and mechanical thermal noises of the accelerometer, thermal noises of resistors, and voltage and current noises of operational amplifier. Figure 2b–h show the circuits for the derivation of the noise gain for each source based on its equivalent small signal circuit analysis. The noise sources are divided into three categories, namely, intrinsic noise sources from the accelerometer, noise sources due to resistors, and noise sources due to the JFET operational amplifier. The detailed steps of finding the individual gains equations are shown in the subsections.

#### 2.2.1. Intrinsic Noise Sources from Accelerometer ($v_{nm}$ and $v_{ne}$) 

Thermal noise in the piezoelectric sensor originates from two sources. The mechanical thermal noise $v_{nm}$ is generated by the mechanical resistance due to the damped harmonic oscillation in the structure. It is given by the following relation [19]:

(12) $$v_{nm}=\sqrt{\frac{4k_{b}T\omega_{o}{Q_{PE}}^{2}}{MQ{C_{PE}}^{2}}}$$

where K_b_ is the Boltzmann’s constant and its value is 1.38 × 10^−23^ J/K, T is the temperature at 300 degrees Kelvin, M is the seismic mass, Q is the quality factor, and ω_o_ represents the resonant frequency in rad/sec. Figure 2b shows the small-signal equivalent circuit to derive the noise gain due to the mechanical thermal noise source of the piezoelectric accelerometer. We define this source as $v_{nm}\;and\;v_{o,nm}$ is designated as the output noise voltage due to $v_{nm}$. In this circuit, all other voltage noise sources in Figure 2a are short-circuited, current noise source is open-circuited, and DC voltage sources are replaced by ground.

Equation (6) can be modified for noise analysis as:

(13) $$(v_{Y}-v_{X})A_{op}=v_{o}$$

With $v_{Y}$ = 0 and $v_{o}=v_{o,nm},$ Equation (13) becomes:

(14) $$v_{X}=-\frac{{\;\;v}_{o,nm}}{A_{op}}$$

KCL at node $v_{X}$ gives:

(15) $$\frac{v_{X}-v_{nm}}{Z_{1}}=-\frac{v_{X}-v_{o,nm}}{Z_{2}}$$

(16) $$⟹v_{X}(\frac{Z_{1}+Z_{2}}{Z_{1}Z_{2}})-\frac{v_{o,nm}}{Z_{2}}=\frac{V_{nm}}{Z_{1}}$$

Using Equation (14) in (16) and rearranging gives the following:

(17) $$G_{nm}=\frac{v_{o,nm}}{v_{nm}}=-\frac{{A_{op}Z}_{2}}{{{(A}_{op}+1)Z}_{1}+Z_{2}}\;$$

The electrical thermal noise ($v_{ne})$ is due to the dielectric losses in the piezoelectric element. It is also known as loss angle or loss tangent and is given by the following relation [19]:

(18) $$v_{ne}=\sqrt{\frac{4k_{b}T\eta }{\omega C_{PE}}}$$

where η is the dissipation factor and ω is the radian frequency. From the equivalent circuit point of view, $v_{ne}$ and $v_{nm}$ are located in the same node, as shown in Figure 1b. Therefore, the same mathematical derivation follows for the noise gain $G_{nm}$ and $G_{ne}$. The corresponding voltage noise gain due to $v_{ne}$ is given as:

(19) $$G_{ne}=\frac{v_{o,ne}}{v_{ne}}=-\frac{{A_{op}Z}_{2}}{{{(A}_{op}+1)Z}_{1}+Z_{2}}$$

where $v_{o,ne}$ is the output noise voltage due to $v_{ne}$. 

#### 2.2.2. Noise Sources Due to Resistors ($v_{nRi},\;v_{nR1},\;v_{nR2},\;and\;v_{nRf}$) 

The thermal noises due to any resistor R is equal to √4K_b_TR. This noise is due to thermal agitation of the electrons within the conductor at equilibrium. Its value depends mainly on the temperature, regardless of applied voltage. Figure 2c shows the small-signal equivalent circuit to derive the noise gain. There are two important parameters. The voltage noises generated by R_i_ are represented by $v_{nRi}$, and $v_{o,Ri}$ is defined as the output noise voltage due to $v_{nRi}$. Other voltage noise sources in Figure 2a are short-circuited, the current noise source is open-circuited, and any DC source is shorted.

We start by modifying Equation (13) to suit Figure 2c with $v_{Y}=0$ and ${v_{o}=v}_{o,Ri}$.

(20) $$(0-v_{X})A_{op}=v_{o,Ri}$$

(21) $$⟹{\;v}_{X}=-\frac{v_{o,Ri}}{A_{op}}$$

The current flowing through $Z_{2}$ is given as follows by Ohm’s law:

(22) $$\;I=\frac{\;v_{o,Ri}-v_{X}}{Z_{2}}$$

The same current (I) flows through R_i_ and C_PE_. By KVL, we have the following equations:

(23) $$-v_{X}+IR_{i}+v_{nRi}+v_{\mathrm{cPE}}=0$$

(24) $$⟹-v_{X}+IR_{i}+v_{nRi}+I{\left(sC_{PE}\right)}^{-1}=0$$

Using Equations (21) and (22) in Equation (24) and rearranging gives the following relation:

(25) $$G_{nRi}=\frac{v_{o,Ri}}{v_{nRi}}=-\frac{s{A_{op}Z}_{2}C_{PE}}{{(A}_{op}+1)+sC_{PE}\{{(A}_{op}+1)R_{i}+Z_{2}\}}$$

Figure 2d shows the equivalent circuit to derive noise gain for R_1_. The voltage noises generated by R_1_ are represented by $v_{nR1}$, while $v_{o,R1}$ is defined as the output noise voltage due to $v_{nR1}$. To derive the noise gain, other voltage noise sources are short-circuited, current noise source is open-circuited, and DC voltage sources are set to 0. 

The voltage $v_{Y}$ in Figure 2d can be obtained from the voltage divider rule as:

(26) $$v_{Y}=\frac{{\left(sC_{2}\right)}^{-1}{||R}_{2}}{{\left(sC_{2}\right)}^{-1}{||R}_{2}+R_{1}}\;v_{nR1}$$

(27) $$⟹\;v_{Y}=\frac{R_{2}}{R_{1}{+R}_{2}+sR_{1}R_{2}C_{2}}v_{nR1}$$

By using the value of $v_{Y}$ from Equation (27) in (13) with $v_{o}=v_{o,R1}$, $v_{X}$ is given as:

(28) $$v_{X}=\frac{R_{2}}{R_{1}{+R}_{2}+sR_{1}R_{2}C_{2}}v_{nR1}-\frac{v_{o,R1}}{A_{op}}$$

Now, KCL at $v_{X}$ gives:

(29) $$\frac{v_{X}}{Z_{1}}=-\frac{v_{X}-v_{o,R1}}{Z_{2}}$$

By using $v_{X}$ from Equation (28) in (29) and rearranging, we obtain the noise gain for R_1_:

(30) $$G_{nR1}=\frac{v_{o,R1}}{v_{nR1}}=\frac{{A_{op}(Z}_{1}+Z_{2})R_{2}}{{[Z}_{1}{(A}_{op}+1)+Z_{2}]({R_{1}+R}_{2}+sR_{1}R_{2}C_{2})}$$

Next, we derive the noise gain equation for R_2_. The equivalent circuit is shown in Figure 2e. The voltage noises generated by R_2_ are represented by $v_{nR2}$ and $v_{o,R2}$ is the output noise voltage due to $v_{nR2}.$ In this equivalent circuit, other voltage noise sources are short-circuited, current noise source is open-circuited, and DC sources are grounded.

The voltage $v_{Y}$ in Figure 2e can be obtained from the voltage divider as below:

(31) $$v_{Y}=\frac{{\left(sC_{2}\right)}^{-1}{||R}_{1}}{{\left(sC_{2}\right)}^{-1}{||R}_{1}+R_{2}}\;v_{nR2}$$

(32) $${⟹\;v}_{Y}=\frac{R_{1}}{R_{1}{+R}_{2}+sR_{1}R_{2}C_{2}}{\;v}_{nR2}$$

By using the value of $v_{Y}$ from Equation (32) in (13) with $v_{o}=v_{o,R2}$, $v_{X}$ is given as:

(33) $$v_{X}=\frac{R_{1}}{R_{1}{+R}_{2}+sR_{1}R_{2}C_{2}}\;v_{nR2}-\frac{v_{o,R2}}{A_{op}}$$

Now, KCL at $v_{X}$ gives:

(34) $$\frac{v_{X}}{Z_{1}}=-\frac{v_{X}-v_{o,R2}}{Z_{2}}$$

By using $v_{X}$ from Equation (33) in (34) and rearranging, we obtain:

(35) $$G_{nR2}=\frac{v_{o,R2}}{v_{nR2}}=\frac{{A_{op}(Z}_{1}+Z_{2})R_{1}}{{[Z}_{1}{(A}_{op}+1)+Z_{2}]({R_{1}+R}_{2}+sR_{1}R_{2}C_{2})}$$

The largest resistor in Figure 1a is R_f,_ and, therefore, its noise contribution will be significant. The equivalent circuit to find this parameter is shown in Figure 2f. The voltage noises generated by R_f_ are represented by $v_{nRf}$, while $v_{o,Rf}$ is the output noise voltage due to $v_{nRf}.$ In this equivalent circuit, we short other voltage noise sources and DC voltage source and set current noise source to equal to 0 A, i.e., open circuit.

Equation (13) can be modified to suit Figure 2f with $v_{Y}=0$ and ${v_{o}=v}_{o,Rf}$ as:

(36) $$(0-v_{X})A_{op}=v_{o,Rf}$$

(37) $$⟹\;v_{X}=-\frac{v_{o,Rf}}{A_{op}}$$

KCL at node $v_{X}$ gives:

(38) $$\frac{v_{X}}{Z_{1}}=-[\frac{v_{X}-v_{o,Rf}}{{\left(sC_{f}\right)}^{-1}}+\frac{v_{X}-(v_{o,Rf}+v_{nRf})}{R_{f}}]$$

(39) $$⟹v_{X}\;\left[\frac{1}{Z_{1}}+\frac{1}{{\left(sC_{f}\right)}^{-1}}+\frac{1}{R_{f}}\;\right]=\frac{v_{o,Rf}}{{\left(sC_{f}\right)}^{-1}}+\frac{v_{o,Rf}}{R_{f}}+\frac{v_{nRf}}{R_{f}}$$

By using the value of $v_{X}$ from Equation (37) in (39) and rearranging, we obtain:

(40) $$G_{nRf}=\frac{v_{o,Rf}}{v_{nRf}}=-\frac{{A_{op}Z}_{1}}{{R_{f+}Z}_{1}{(A}_{op}+1)(1+sR_{f}C_{f})}$$

#### 2.2.3. Noise Sources Due to the JFET Operational Amplifier ($v_{nop}\;and\;i_{nop}$)

A JFET operational amplifier has two noise sources: voltage noise ($v_{nop}$) and current noise ($i_{nop}$). From the LT1169 datasheet [18], the value of $v_{nop}$ is 6nV/√Hz. We calculate $i_{nop}$ from the equation √(2qI_b_), where q is the electronic charge (1.6 × 10^−19^ C) and I_b_ is the input bias current (1.5 pA).

Figure 2g shows the equivalent circuit to derive the noise gain due to $v_{nop}$. In this circuit, we set other voltage noise sources in Figure 2a equal to 0 V, while current noise source is open-circuited. In addition, all DC sources are set to 0 V. Given that $v_{o,vnop}$ is the output noise voltage due to $v_{nop},$ the corresponding noise gain equation is derived as follows:

First of all, Equation (13) can be modified for Figure 2g as follows with $v_{Y}=0$ and ${v_{o}=v}_{o,vnop}$:

(41) $$(0-v_{X})A_{op}=v_{o,vnop}$$

(42) $$⟹\;v_{X}=-\frac{v_{o,vnop}}{A_{op}}$$

KCL at node A gives:

(43) $$\frac{v_{X}+v_{nop}}{Z_{1}}=-\frac{{(v}_{X}+v_{nop})-v_{o,vnop}}{Z_{2}}$$

(44) $$⟹v_{X}\;(\frac{Z_{1}+Z_{2}}{Z_{1}})+v_{nop}\left(\frac{Z_{1}+Z_{2}}{Z_{1}}\right)=v_{o,vnop}$$

By using the value of $v_{X}$ from Equation (42) in (44) and rearranging, we obtain the noise gain:

(45) $$G_{nop}=\frac{v_{o,vnop}}{v_{nop}}=\frac{{A_{op}(Z}_{1}+Z_{2})}{{(A}_{op}+1)Z_{1}+Z_{2}}$$

The final noise source in Figure 2a is called $i_{nop}$, which is the current noise of the operational amplifier. The equivalent circuit is shown in Figure 2h. We define $v_{o,inop}$ as the output noise voltage due to $i_{nop}.$ In order to derive the noise gain, other voltage noise sources in Figure 2a as well as all DC voltage supplies are short-circuited.

Equation (13) can be modified per Figure 2h by setting ${v_{o}=v}_{o,inop}$.

(46) $${(v}_{Y}-v_{X})A_{op}=v_{o,inop}$$

(47) $$⟹\;v_{X}{=v}_{Y}-\frac{v_{o,inop}}{A_{op}}$$

The value of $v_{Y}$ is given as follows:

(48) $$v_{Y}={\{\left(sC_{2}\right)}^{-1}{||R}_{1}{||R}_{2}\}\;i_{nop}$$

(49) $$⟹\;v_{Y}=\frac{R_{1}R_{2}}{R_{1}{+R}_{2}+sR_{1}R_{2}C_{2}}\;i_{nop}$$

By using the value of $v_{Y}$ from Equation (49) in (47), $v_{X}$ is given as follows:

(50) $$v_{X}=\frac{R_{1}R_{2}}{R_{1}{+R}_{2}+sR_{1}R_{2}C_{2}}\;i_{nop}-\frac{v_{o,inop}}{A_{op}}$$

KCL at node $v_{X}$ gives:

(51) $$i_{nop}=\;\frac{0-v_{X}}{Z_{1}}+\frac{v_{o,inop}{-v}_{X}}{Z_{2}}$$

By using the value of $v_{X}$ from Equation (50) in (51) and rearranging, we obtain:

(52) $$G_{inop}=\frac{v_{o,inop}}{i_{nop}}=\frac{{A_{op}[(R_{1}{+R}_{2}+sR_{1}R_{2}C_{2})Z}_{1}Z_{2}+R_{1}R_{2}(Z_{1}+Z_{2})]}{\left[R_{1}{+R}_{2}+sR_{1}R_{2}C_{2}\right][{(A}_{op}+1)Z_{1}+Z_{2}]}$$

## 3. Results and Discussion

**Figure 3:**
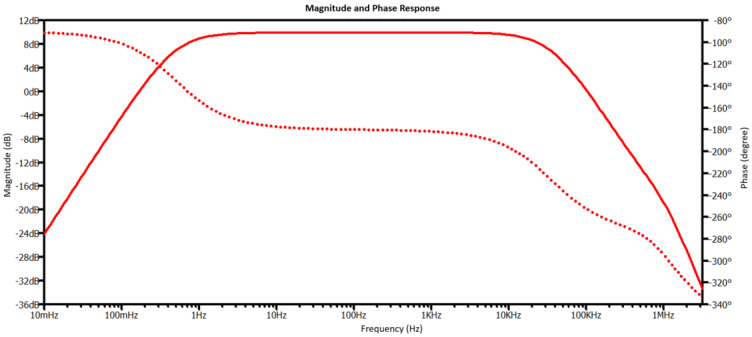
The voltage gain (in magnitude (solid line) and phase response (dotted line)) of the system.

Figure 3 shows the voltage gain of the circuit in Figure 1a, which is simulated from LTSpice. The amplitude and the phase are represented by the bold and dotted lines, respectively. There are a couple of observations from the plot of the magnitude. First, the flat gain of 10 dB can be observed, indicating a linear response. Second, the −3 db lower cutoff frequency of 0.49 Hz meets the requirement for the lowest operating requirement of the accelerometer. Third, the −3 db upper cutoff frequency is around 35 KHz, which is way above the accelerometer’s upper frequency range of 10 KHz. We over-engineer this upper frequency limit due to R_i_, since we choose a small value for this resistor to minimize the amplifier noise. Finally, the phase response plot shows consistent inverting amplifier characteristics.

**Figure 4:**
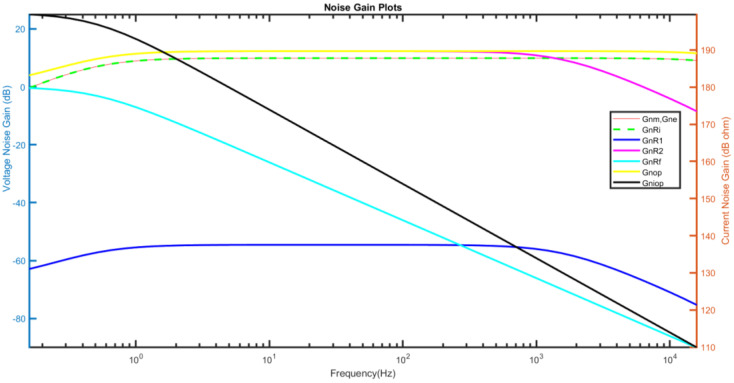
Noise gain from individual sources.

Figure 4 shows the plots of noise gains for all sources across the operating frequency of the accelerometer. MATLAB is used to plot Equations (17), (19), (25), (30), (35), (40), (45), and (52). The left y-axis shows the values of voltage noise gain, which is applicable for $v_{nm},\;v_{ne},\;v_{nRi},\;v_{nR1},\;v_{nR2},\;v_{nRf},\;and\;v_{nop}$. The right y-axis shows the value of the current noise gain for $i_{nop}$. Since the noise gains of the electrical thermal noise and mechanical thermal noise are identical, both are presented by the same red line in Figure 4.

**Figure 5:**
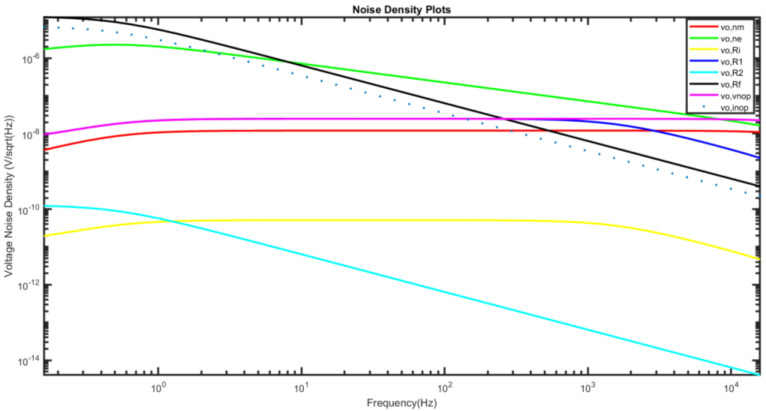
Noise density from individual sources.

Figure 5 shows the noise densities of individual sources. This very important parameter is obtained by multiplying the individual noise gain with its corresponding voltage or current source. There are several important observations from Figure 5. First, the noise of R_f_ dominates at the lower frequency, as the value of R_f_ is set to 10 GΩ to push the lower cutoff frequency below 0.5 Hz. Second, the electrical thermal noise of the accelerometer dominates after 10.28 Hz. Third, the lowest noise density is from R_2_, as its value is only 1 Ω.

Based on the superposition principle, the total noise density at the output node is the geometric sum of the individual noise densities. This is illustrated in Equation (53). In order for this relationship to hold, we assume that there is no correlation between all the noise sources [10,11].

(53) $$v_{o}=\sqrt{\sum_{x\in v}v_{0,x}^{2}}$$

where $v=\{v_{nm},v_{ne},v_{nRi},v_{nR1},v_{nR2},v_{nRf},v_{nop},i_{nop}\}$.

**Figure 6:**
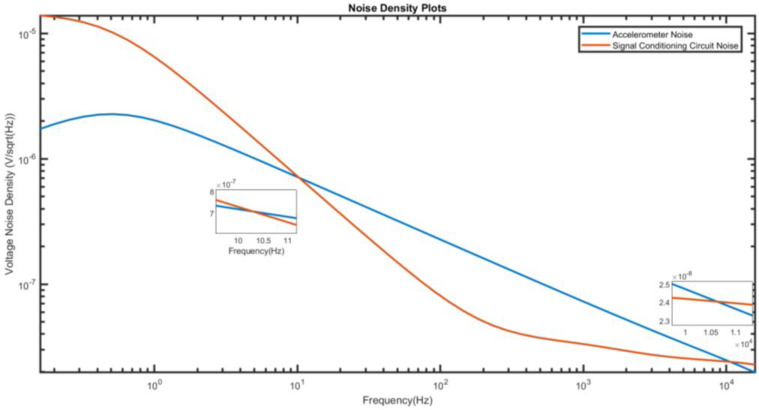
Total noise density from accelerometer and signal conditioning circuit.

We use Equation (53) to separately calculate the total noise density of the piezoelectric accelerometer, as well as the total noise density of the charge amplifier. This information is useful to determine which one dominates in this system. The result is shown in Figure 6. The noise from the signal conditioning circuit dominates at low frequencies up to 10.28 Hz, while the noise from the sensor is higher after that point. As a reference, the piezoelectric accelerometer’s operating bandwidth is from 0.5 Hz to 10 kHz.

## 4. Practical Considerations

We would like to offer a couple of practical considerations to make this model useful. The proposed model can serve two different classes of people. The first is the designers of the piezoelectric accelerometer who want to figure out the most suitable signal conditioning circuit. They have the full information of their devices such as the proof mass, quality factor, capacitance, etc., to model the intrinsic noise sources of their designed accelerometer. The second is the users of the commercial piezoelectric accelerometer who do not have access to the device parameters, as these are considered trade secrets by the companies. The authors belong to this group. Since this group could not make use of Equations (12) and (18) to find the values of mechanical- and electrical-thermal noise densities, our recommendation is to read the product datasheet to obtain information on the noise density (commonly referred to as noise floor). The modeling of the noise of the signal conditioning circuit remains the same and can be combined with the noise floor of the accelerometer to make a meaningful analysis that is shown in Figure 6.

We would also like to add practical recommendations to reduce the noise density from the signal conditioning circuit. In terms of circuit topology, the simpler the circuit, the less the component counts and, hence, the less noise sources that could add up. This is why we choose the basic charge amplifier configuration to pair it with the piezoelectric accelerometer. The selection of the components also plays a critical role in reducing their noise density. Due to the imperfection in the manufacturing processes, each component contains parasitic elements [20]. Any capacitor will have parasitic resistance and inductance. The parasitic resistance will contribute additional thermal noise, while the parasitic inductance will pick up noise from the external magnetic signals. We recommend the use of a tantalum capacitor for low-frequency noise measurement. In addition, the type of resistor must also be carefully considered. We should avoid using the composition-type resistor as it generates contact noise, since it is made of many particles molded together. The contact noise produced by the film-type resistors is much less than that produced by composition resistors because the material is more homogeneous. Hence, the former is recommended in the signal conditioning circuit, especially for the ones with large values such as R_f_.

The third recommendation regards the selection of the signal conditioning circuit. In this work, the basic JFET charge amplifier circuit has provided sufficient voltage gain (10 dB) and bandwidth (35 kHz) for the chosen piezoelectric accelerometer. However, this basic circuit may not be suitable for other piezoelectric accelerometers [21] because some applications require voltage scaling and conversion from analog to digital signals. Furthermore, some sensors require impedance matching with its signal conditioning circuit [22]. All these extra functionalities point to the need for a more complicated signal conditioning circuit.

## 5. Conclusions

This paper performs comprehensive noise modeling of a piezoelectric charge accelerometer with a signal conditioning circuit. This is accomplished by identifying all noise sources in the system. After that, we perform small-signal equivalent circuit analysis to derive the noise gain for each source. Finally, the more familiar noise density values are plotted for meaningful analysis. While this method has been reported by Durdaut [11] for a magnetoelectric sensor, we are applying it on a piezoelectric charge accelerometer with JFET operational amplifier.

After completing the analysis of the proposed model, we are of the opinion that modern JFET operational amplifiers have potential to replace multistage discreet amplifiers consisting of JFET, MOSFET, and BJT. This is largely attributed to advances being made by manufacturers to produce operational amplifiers with ultra-low noise, high input impedance, and large open-loop gain. In this paper, this is demonstrated using a piezoelectric accelerometer that is paired with LT1169 to serve as a basic charge amplifier circuit. As shown in Figure 6, the noise density from the signal conditioning circuit only dominates at low frequency up to 10.28 Hz. As a comparison, Levinzon also reported the corner frequency of 10 Hz for the noise spectral density of JFET input stage [11], which contributed to 1/*f* noise of that transistor.

## Figures and Tables

**Table 1:** Parameters of the piezoelectric charge accelerometer [19].

Parameter	Value
Resonant frequency ‘f_o_’	30 KHz
Mass ‘M’	3 × 10^−4^ Kg
Quality factor ‘Q’	70
Charge Sensitivity ‘Q_PE_’	1 pC/g
Capacitance ‘C_PE_’	100 pF
Dissipation factor ‘η’	0.02
Temperature ‘T’	300 K
Operating frequency	0.5 Hz to 10 KHz

**Table 2:** Parameters of the signal conditioning circuit.

Parameter	Value
R_i_	45 KΩ
R_1_	2.2 KΩ
R_2_	1 Ω
C_f_	32 pF
R_f_	10 GΩ
C_2_	100 uF
V+	5 V
V-	−5 V
Aop	1.2 × 10^6^

## Data Availability

The data that support the findings of this study are available from the authors upon reasonable request.
